# Efficacy and Safety of Intravenous Immunoglobulin Treatment in Selected Neurological Diseases—One Centre’s Experience Based on the Therapy of 141 Patients

**DOI:** 10.3390/jcm12185983

**Published:** 2023-09-15

**Authors:** Anetta Lasek-Bal, Anna Wagner-Kusz, Barbara Rogoż, Małgorzata Cisowska-Babraj, Gabriela Gajewska

**Affiliations:** 1 Department of Neurology, School of Health Sciences, Medical University of Silesia in Katowice, Ziołowa Str. 45/47, 40-735 Katowice, Poland; annawag@onet.pl; 2 Department of Neurology, Upper-Silesian Medical Centre of the Silesian Medical University in Katowice, 40-735 Katowice, Poland; tinar@o2.pl (B.R.); neurologia@gcm.pl (M.C.-B.); modzel247@wp.pl (G.G.)

**Keywords:** immunoglobulin, IVIg, myasthenia gravis, Guillain–Barré syndrome, autoimmune encephalitis, NMOSD, CIDP

## Abstract

Background: Intravenous immunoglobulins (IVIg) are the first-choice drugs for the treatment of certain neuroimmune diseases. The aim of this study was to evaluate the efficacy and safety of IVIg in patients with selected nervous system diseases. Methods: The study enrolled patients who received IVIg in programmes financed by the National Health Fund in Poland. The status of patients upon inclusion and during treatment was assessed using scales dedicated to specific neurological diseases. Results: The study enrolled 141 patients aged 56.28 ± 14.72 (51.77% female): 21 patients with myasthenia gravis (MG), 65 with chronic inflammatory demyelinating polyneuropathy (CIDP), 30 with Guillain–Barré syndrome (GBS), 12 with neuromyelitis optica spectrum disorder (NMOSD) and 13 patients with autoimmune encephalitis (AE). Neurological improvement was found in 14 (66.66%) MG patients (with a reduction of at least three points on the Quantitative Myasthenia Gravis Score (QMGS) within 14 days from the completion of the cycle), and in 34 (52.3%) GBS patients (with a reduction of at least one point on the Medical Research Council Scale within 14 days from the completion of the cycle). The parameters with the strongest effect on clinical improvement in MG patients were age [OR 1.033, CI 95% [0.09–1.09], *p* = 0.049] and baseline QMGS [OR 0.505; CI 95% [0.24–0.87], *p* = 0.038]. In the majority of CIDP patients (27, 97%) and NMOSD patients (6, 50%), neurological stabilisation was observed (without clinical improvement, defined for CIDP patients as an increase of at least two points on the Lovett Scale after three courses of IVIg were administered, and for NMOSD patients as an increase of at least one point on the Medical Research Council Scale and/or a shift of at least 0.3 logMAR after three courses of treatment). Deep-vein thrombosis was only one serious adverse event in the total group of patients treated with IVIg. Conclusions: The use of IVIg in patients with MG and GBS mostly results in neurological improvement, while in patients with NMOSD and CIDP, it mostly results in disease stabilisation. This could indicate the predominant anti-idiotypic antibody activity of IVIg in acute neuroimmune diseases or during exacerbations in chronic autoimmune diseases. The therapy of AE in comorbid neoplastic disease is burdened with an elevated risk of failure for IVIg. The results of our study confirm the improved safety of IVIg for selected neurological diseases.

## 1. Introduction

Autoimmune diseases occur because of an abnormal immune response towards self-structures due to loss of self-tolerance. This abnormal response can be directed against the structures of the central or peripheral nervous system; it can also be directed against neuromuscular junctions. Autoimmune diseases are characterised by an inflammatory process, the production of antibodies and cytokines, and tissue damage. The relevant therapy involves interference with the immune system, i.e., the elimination of antibodies from both cellular and soluble components. One of these therapeutic methods, intravenous immunoglobulin (IVIg) application, has been successfully used in single or repeatable cycles, depending on the type of disease. The effect of IVIg is achieved through several mechanisms: anti-idiotypic antibody activity, saturation of neonatal Fc receptors (FcRn), upregulation of inhibitory FcγRIIb receptors and inhibition of macrophage activation, anti-complement activity, and downregulation of cofactors and adhesion molecules [1].

As recommended by experts today, IVIg are the first-choice drugs for the treatment of the deterioration associated with and/or in the course of certain acute and chronic neuroimmune diseases, e.g., myasthenia gravis (MG), chronic inflammatory demyelinating polyradiculoneuropathy (CIDP), Guillain–Barré syndrome (GBS), neuromyelitis optica spectrum disorder (NMOSD) and autoimmune encephalitis (AE). However, evidence regarding the efficacy of IVIg in some neurological autoimmune diseases is still limited and/or the therapeutic effects of IVIg are inconsistent.

The application of IVIg during periods of MG exacerbation has been found to produce effects equivalent to plasma exchange (PE), yet with fewer adverse effects [2]. IVIg drugs are also used in chronic cyclical therapy for CIDP. Like glucocorticosteroids, they are used as a first-line treatment. They are also the first-choice drugs in the treatment of GBS, as they have repeatedly shown efficacy comparable to PE but with better tolerability. IVIg are used in patients with NMOSD when immunosuppressive drugs fail or are poorly tolerated. In addition to glucocorticosteroids, IVIg drugs have also been used in the treatment of AE. The efficacy of IVIg in the above-mentioned conditions is due to their anti-inflammatory and immunomodulatory properties.

The aim of this study was to evaluate, among our 141 patients, the efficacy and safety of IVIg in patients with MG, GBS, CIDP, NMOSD and AE. Another aim of the present study was to identify the factors that played an important role in achieving a favourable clinical effect for IVIg therapy in our patients.

## 2. Methods

### 2.1. Patients

This real-world study enrolled patients who received IVIg as part of various drug programs at the Department of Neurology at The Upper Silesian Medical Centre, The Medical University of Silesia in Katowice, between 2019 and 2021. The sociodemographic and clinical data of patients who met the inclusion criteria for the B.67 Drug Programme and were treated according to the programme’s guidelines, “Immunoglobulin treatment in neurological diseases” [B.67] were collected.

### 2.2. Clinical Assessment

For the purposes of the study, the status of each patient was assessed using scales dedicated to specific neurological conditions, i.e., the Quantitative Myasthenia Gravis Score—QMGS (MG), Lovett (CIDP), Medical Research Council—MRC (NMOSD, GBS) and mRS—modified Rankin Scale (AE). An ophthalmologist’s examination (MAR) was also used to evaluate the patients with NMOSD. The safety assessment included an analysis of any adverse events resulting from the products’ side-effects and the reasons for any unscheduled discontinuation of therapy. Patients were also monitored for potential adverse events identified in the product characteristics as complications of the therapy. For the purposes of the study, “chronic immunosuppressive therapy” was defined as the oral use of steroids or other immunosuppressive drugs for at least six months prior to IVIg and during IVIg enrolment. All patients were clinically monitored within the scope of 12 months, with a neurological/functional assessment 2 weeks after 1 initial IVIg cycle (MG and GBS patients) or after 3 IVIg cycles (the other patients).

For the purpose of this study, the definitions of clinical improvement/stabilisation were established according to the principles of therapy in the B67 programme, the authors’ own experience, as well as the experience of the other researchers [3,4].

Below are the definitions of neurological improvement in the course of diseases evaluated in the study.

Neurological improvement in patients with MG was defined as a reduction of at least 3 points on the QMGS within 14 days from completion of the therapy [5].

Neurological improvement in patients with CIDP was defined as an increase of at least 2 points on the Lovett Scale after 3 cycles of IVIg were administered.

Lovett scale: grade 0—no evidence of contractility; grade 1—slight contractility without movement; grade 2—full range of motion when gravity has been eliminated; grade 3—full range of motion with gravity; grade 4—full range of motion with gravity, some resistance; grade 5—full range of motion with gravity, full resistance, normal.

Neurological improvement in GBS patients was defined as a reduction of at least 1 point on the MRC Scale within 14 days from completion of cycle.

Medical Research Council: grade 5—muscle contracts against full resistance; 4—strength reduced but contraction can still move joints against resistance; 3—strength further reduced so that joint can be moved only against gravity with examiner’s resistance completely removed; 2—muscle can only move if resistance of gravity is removed; 1—only a trace or flicker of movement is seen/felt or fasciculations are observed; and 0—no movement.

Neurological improvement in patients with NMOSD was defined as an increase by at least 1 point on the MRC Scale and/or a shift by at least 0.3 logMAR after 3 courses of treatment.

(LogMAR chart—the Logarithm of the Minimum Angle of Resolution—consists of rows of letters used by ophthalmologists to estimate visual acuity; logMAR score 0.3—when patient can resolve details as small as 2 min of visual angle).

In our opinion, based on our experience, the effect of treatment in patients with AE could be defined as good if a patient’s functional status of 0–2 on the mRS was achieved after at least 3 courses of treatment, or as moderate if there was a decrease of at least 1 point on the mRS in patients in bad functional state (3–5 mRS) upon initiation of therapy.

The modified Rankin Scale is as follows: 0—no symptoms; 1—no significant disability, able to carry out all usual activities despite some symptoms; 2—slight disability, able to look after oneself without assistance but unable to carry out all previous activities; 3—moderate disability, requires some help but able to walk unassisted; 4—moderately severe disability, unable to attend to one’s own bodily needs without assistance and unable to walk unassisted; 5—severe disability, requires constant nursing care and attention, bedridden, incontinent; and 6—dead.

Lack of clinical improvement (according to the above-mentioned definitions) was assessed as the stabilisation of clinical status. Each unfavourable change in the clinical (neurological) condition was assessed as clinical deterioration.

Neurological examinations and assessments according to clinical scales were performed by 2 neurologists experienced in IVIg therapy.

### 2.3. Statistical Analysis

To assess the mean and median values of the medical data, descriptive statistical analysis was conducted.

In some groups (MG, CIDP and GBS patients), uni- and multivariate analyses were performed to identify the factors which were important for a good clinical outcome (improvement in MG, GBS or at least stabilisation of CIDP).

Multivariable models were built using ordinal logistic regression for ordinal outcomes. The variable selection procedures for these models included automatic selection (stepwise, forward and backward) based on AIC and BIC criteria. To evaluate the accuracy of the model predictions, the “leave one out” procedure and the multiclass AUC estimator were used. All statistical analyses were performed using R version 3.6.1.

The following parameters were analysed with logistic regression: age, sex, disease duration before IVIg treatment, previous chronic therapy, dose/kg bw, dose/course, intervals between courses, total dose (the entire IVIg therapy) and clinical status as per the related scales.

Since the study was not a medical experiment, the Bioethics Committee of the Medical University of Silesia in Katowice was not required to provide an opinion.

## 3. Results

The study enrolled 141 patients (of which 73, 51.77%, were female) treated with IVIg as part of drug programmes, including 21 patients with MG, 65 with GBS, 30 with CIDP, 12 with NMOSD and 13 with AE. A general breakdown of the patients is presented in Table 1.

### 3.1. MG

The study enrolled 21 patients with MG at a mean age of 56.71 ± 21.22, med. 66 [28–88] and a mean disease duration of 6.13 years [0.5–18]. Of these, 10 patients had been treated previously with oral immunosuppression using one drug (steroid), six with at least two drugs, and five patients had not been subjected to any chronic immunosuppressive therapy. The mean duration of IVIg therapy in the B.67 Drug Programme was 4.54 months, med. 3 [1–21]. The range of doses used was 1.0–2.25 g/kg bw (mean 1.55 g/kg bw, mean dose/course 207.38 g). The average interval between doses was 22.80 days [37–116]. The following IVIg preparations were used: NanoGy in three patients, IgVena in five patients, Privigen in six patients, Sandoglobulin in one patient, and two preparations, IgVena followed by Kiovig, were used in one patient; Kiovig and NanGy were used in one patient; IgVena was used in four patients. Neurological improvement was found in 14 patients (66.66%), stabilisation was found in 6 patients (28.57%), and deterioration was found in 1 patient. Parameters with the strongest effect on clinical improvement were age [OR 1.033, CI 95% [0.09–1.09], *p* = 0.049] and baseline QMGS [OR 0.505; CI 95% [0.24–0.87], *p* = 0.038]. The younger age (<50) as well as grade ≤2 in QMGS (QMG ≤ 16) were positively correlated with the probability of clinical improvement during assessment performed 14 days after IVIg infusion.

Deep-vein thrombosis (DVT, in leg) occurred in one patient on day seven after the initiation of therapy and a transient rash was found in one patient on the second day of treatment. The therapy was discontinued in one patient due to DVT.

### 3.2. GBS

The study enrolled 65 patients with GBS at a mean age of 52.75 ± 14.13, med. 56 [19–77]. The mean duration of the disease was 14.70 days [2–30]. In 27 patients, IVIg was initiated in the first week of onset. A single 5-day cycle (dose 2.0/kg bw) was administered using the following preparations: Privigen in 21 patients, IgVena in 28 patients, Kiovig in 6 patients, and IgVena in 10 patients. An improvement was observed in 34 patients (52.3%), and 20 patients showed stabilisation (30.76%). We did not identify any parameters important for clinical improvement in the univariable model. The neurological status of other patients deteriorated over time, including three patients who developed respiratory failure requiring mechanical ventilation therapy. In one patient, tachycardia, sweating, and restlessness presented during the intravenous infusion of IVIg (on the first day); therefore, drug infusion was discontinued. On the following day, symptoms of this nature did not occur, and the patient was able to continue therapy.

### 3.3. CIDP

The study enrolled 30 patients with CIDP at a mean age of 60.37 ± 13.86, med. 61 [29–79]. The mean disease duration at enrolment was 6.4 years, med. 5.5 [1–17]. Before IVIg administration, 12 patients had been taking one immunosuppressive drug and the other 18 at least two immunosuppressive drugs. The range of IVIg doses used was 1.0–2.0 g/kg bw (mean 1.28 g/kg bw). The therapy duration was as follows: mean 23.75 months [1–69]. The mean interval between doses was 69.3 days [0–116]. The following preparations were administered: Privigen in 10 patients, IgVena in 7 patients; two preparations, NanoGy followed by IgVena, were used in 7 patients, Kiovig and NanGy were used in 2 patients, Kiovig and Provigen were used in 2 patients, and Kiovig and IgVena were used in 2 patients. Improvement was observed in 3 patients (1%); stabilisation was observed in the other 27 (97%) patients. There were no parameters displaying the strongest correlation with clinical stabilisation. There were no side-effects of IVIg therapy according to the product characteristics in this patient group.

### 3.4. MNOSD

The study enrolled 12 patients with NMOSD at a mean age of 49.75 ± 8.91, med. 49.5 [27–78]. The mean duration of the disease (from the moment of diagnosis) was 4.17 years, med. 3 [1–11] Before IVIg administration, four patients had been put on chronic immunosuppressive therapy with one drug, seven patients with at least two drugs (not simultaneously); due to poor tolerance (leukopenia), and one patient had not used oral immunosuppression. All patients received an initiation dose of IVIgs at 2.0 g/kg bw and maintenance therapy at 1.0–2.0 g/kg bw, mostly at 2.0 g/kg bw. The mean interval between doses was 49 ± 15.28, med. 47 days [29–69], which was related to changes in the availability of some IVIg on the Polish market. The mean duration of IVIg therapy was 14.91 months ± 4.15, med. 13.5 [4–24]. The following preparations were used: IgVena in four female patients; Privigen in four, NanoGy in one, and Sandoglobulin in one patient; two preparations, IgVena followed by Kiovig, were used in two patients. An improvement was found in three female patients (25%), stabilisation occurred in six patients (50%) and deterioration was apparent in two individuals. Nine female patients (75%) reported a noticeable improvement in visual acuity, but only two cases met the criteria adopted in the study. No female patients reported any adverse effects from the therapy.

### 3.5. AE

The study enrolled 13 patients with AE at a mean age of 61.85 ± 15.48, med. 66 [23–79]. The mean duration of the disease at the time of enrolment was 5.5 months [2.5–60]. Before IVIg was administered, one patient had been on one immunosuppressive drug. Over the course of the disease, 5 patients had epileptic seizures; all had quantitative disturbances of consciousness; qualitative disturbances of consciousness were reported in 11 patients; disorientation and memory disturbances occurred in 9 subjects. MRI showed changes in the temporal lobes of five patients. Antibodies were present in eight patients (NMDA antibodies were found in two patients, AMPA antibodies were found in two patients, LGI-1 antibodies were found in four patients), and onconeuronal antibodies were found in four patients.

The range of IVIg doses used during a single course of therapy were as follows: mean 90 g [50–160]; 1.0–2.0 g/kg bw (mean 1.18 g/kg bw). The mean duration of IVIg therapy was 69.69 days [59–304]. The mean interval between doses was 61 days [36–156]. The following IVIg were used: Privigen in eight patients, IgVena in two patients; Privigen, followed by Kiovig, was used in three patients. In four patients, a neoplastic disease was discovered during the course of diagnosis; oncology therapy was started. IVIg therapy showed a good effect in four patients, a satisfactory outcome in four (stabilisation of functional status) and deterioration in others, including the deaths of three patients. All patients with neoplastic disease (ovarian cancer in three female patients, intestinal neuroendocrine cancer) experienced functional deterioration. One patient had skin lesions of moderate severity.

**Figure 1:**
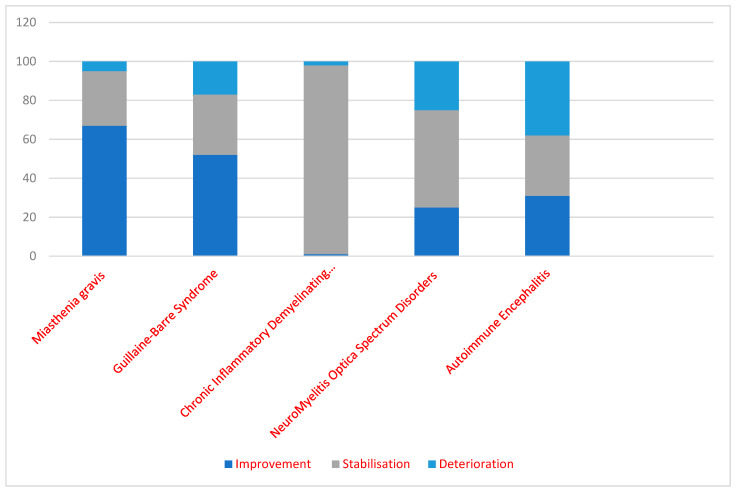
The clinical/functional IVIg therapeutic effect—percentage distribution. (Chronic Inflammatory Demyelinating… = Chronic Inflammatory Demyelinating Polyneuropathy).

A data summary regarding the therapeutic effects of IVIg upon our patients is presented in Figure 1.

## 4. Discussion

The most important outcome of the study is the conclusion that, among patients with selected neuroimmune disorders, patients with GBS and MG benefited the most from IVIg therapy. Clinical stabilisation was most often achieved in patients with NMOSD and CIDP. Human IVIg drugs are well tolerated and seem to be safe in chronic therapy. Although the mechanism of action of IVIg drugs on the nervous system is not fully understood, they appear to have multidirectional, immunomodulatory, and anti-inflammatory effects. They influence both soluble mediators and targets in the immune system. They act as inhibitors of Fc-gamma-RI and Fc-γ-RIII receptors and/or as activators of Fc-γ-RII receptors. They also inhibit macrophage activity, phagocytosis, and macrophage-mediated demyelination. IVIgs also interact with adhesion cells (ICAM, VCAM). They also modulate the endothelial cell function and the effect of the following cytokines: IL-1beta, IL-2, IL-6, IFN-gamma, and B-cell activating factor (BAFF) [1]. Based on our findings, it seems that the most clinically effective mechanism of action in IVIg is the neutralisation of autoantibodies. Perhaps this is why the effect of IVIg is poorer when a degenerative process exists parallel to the inflammatory process, as in demyelinating diseases.

MG is approached with chronic immunosuppression, including corticosteroids as the drugs of first choice. The severity of the disease and steroid therapy are the main factors which worsen the quality of life in MG patients [6]. During periods of disease exacerbation, plasmapheresis or IVIg therapy is used [7]. The effect of IVIg in MG therapy is attributed to autoantibodies being neutralised through anti-idiotype antibodies acting against acetylcholine receptors. This may explain why the effect of a single cycle is depleted. According to current MG treatment guidelines, IVIg should be used in periods of disease exacerbation and myasthenic crisis (as rescue treatment). The results of randomised trials indicate that the use of IVIg in patients with MG at a dose of 1–2 g/kg bw over 2–5 days results in clinical-status improvement and further stabilisation of the disease. As suggested by other authors, cyclical use of a maintenance dose at 1.0 g/kg bw every 3–4 weeks generally improves the neurological status of patients. However, 2–3 weeks after IVIg was administered, patients reported a noticeable decrease in limb-muscle strength. The presented results show the long-term benefits of IVIg in patients with disease exacerbation. In the vast majority of cases, patients with MG achieved neurological improvement after IVIg treatment. In our study, age and QMGS had the strongest effect on neurological improvement. Katzberg described the baseline QMGS predictive of IVIg but not of PE response [8].

We observed a similar and relatively timely effect after IVIg treatment in patients with GBS. The results of randomised trials indicate that the benefits of IVIg therapy are comparable to those observed after plasmapheresis. The therapy should be started in the early stages of the disease, before irreversible axonal damage occurs. The recommended dose of IVIg in GBS patients is 2.0 g/kg bw administered for 5 days. A total of 41% of our patients initiated therapy in the first week of the disease. In our study, we observed the highest percentage of neurological improvement in GBS patients. The other authors observed comparably favourable effects after both therapies using IVIg and PE [9].

To date, no evidence has been found for the efficacy of a second IVIg cycle in GBS patients with a poor prognosis [10].

Intravenous steroid therapy is the standard of care in the treatment of NMOSD relapses. However, consistent results of numerous studies indicate that improvement is short-term, and steroids fail to prevent optic atrophy. Chronic management of patients suffering recurrent relapses includes rituximab, IVIg, plasmapheresis, mycophenolate mofetil, azathioprine and, recently, also satralizumab. Administering IVIg can have a potential role in treating disease exacerbation and an effect on steroid-resistant patients. However, there is not much evidence of the benefits of IVIg therapy. In a retrospective study of 10 patients unresponsive to intravenous steroid infusion, IVIg improved the condition in half of the subjects [11].

Among patients with NMOSD, as well as with CIDP, no more than disease stabilisation was most often observed. The potential role of long-term intermittent IVIg therapy to prevent relapse in NMOSD has not been established. One study showed a decrease in the number of relapses per year and an improvement in the EDSS score in patients treated over a mean period of 19 months [12]. The use of IVIg can be considered as a safe alternative treatment in NMOSD for patients with recurrent infections due to immunosuppressive therapy [13]. The inflammation that develops in NMOSD can lead to demyelination, reactive gliosis, and axonal damage [14]. It is likely that at the time of IVIg inclusion, axonopathy, which causes irreversible structural damage to the nervous system, was present in our patients. It is worth stressing that after IVIg was administered, 3/4 of the patients reported an improvement in visual acuity, which was mostly a subjective finding. Some authors presented similar findings in NMOSD patients who were unresponsive to corticosteroids [15].

CIDP causes moderate to severe motor deficits in most patients. Steroids, other immunosuppressants, IVIg and immunoglobulins administered subcutaneously have a proven effect. Due to a relatively rapid onset of action and fewer side-effects compared to steroids and plasmapheresis, IVIg are often the first-choice drugs [16,17]. Most patients need long-term maintenance treatment to prevent (secondary) axonal damage [18]. RCT results indicate that IVIg therapy, at a total dose of 2.0 g/kg bw administered for 5 days, improves muscle strength over a few weeks. The dosage, intervals and duration of IVIg treatment vary from patient to patient [19]. As shown by the presented results, only 1% of patients exhibited clinical improvement and most subjects showed only stabilisation. All our patients used immunosuppression before IVIg and they probably tended to have a lower degree of baseline disability during IVIg therapy qualification. This relatively lower degree of neurological deficit potentially leaves fewer chances for clinical improvement (“ceiling effect”).

Other authors reported no IVIg efficacy in about a quarter of patients with CIDP [19]. It is suggested that genetic factors underlie the heterogeneity of treatment response in CIDP patients [19,20].

Autoimmune encephalitis is characterised by neuropsychiatric disorders. These include cognitive impairment, behavioural disorders, quantitative disturbances of consciousness, epileptic seizures, and focal deficits [21]. The presence of antibodies against neuronal cell-surface proteins, ion channels or receptors is observed in most patients [22]. The treatment aims to neutralise or eliminate the antibodies through the application of steroids, plasmapheresis or IVIg. Steroids are often administered in combination with plasmapheresis or IVIg [23,24]. Our study results suggest that the effect of IVIg therapy in AE is at best moderate. Other authors observed improvements in 44% of AE patients within 4 weeks of IVIg therapy combined with corticosteroids [25].

Our experience indicates that IVIg can be beneficial as first-line, second-line or adjuvant therapy in selected autoimmune diseases.

## 5. Limitations

Our study has some limitations, as follows. Firstly, the intervals between drug administrations were, in some cases, different from what had been originally planned (due to temporary unavailability of IVIg in Poland). Furthermore, alternative therapies were necessary, with some patients receiving more than one type of medication. The lack of analysis related to a potential impact of comorbidity and parallel chronic treatment of other diseases is another limitation.

## 6. Future Perspectives

Additional studies are necessary to enhance our comprehension of the variations in response to IVIg therapy among patients, as well as to optimise the IVIg treatment with the objective of preventing disease activity and averting irreversible axonal damage.

## 7. Conclusions

The use of IVIg in patients with MG and GBS mostly results in neurological improvement, while in patients with NMOSD and CIDP, it mostly results in disease stabilisation.

This is indicative of the predominant anti-idiotypic antibody activity of IVIg in acute neuroimmune diseases or during exacerbations in chronic autoimmune diseases.

The treatment of AE in comorbid neoplastic disease is burdened with an elevated risk of failure for IVIg therapy.

The results of our study confirm an improved tolerability and safety in human immunoglobulin therapy for selected nervous system diseases.

## Figures and Tables

**Table 1:** The characteristic of patients enrolled in the study.

Disease	Number of Patients, n	Age (Years), Mean ± SD	Sex F/M	Disease Duration Mean	Patient Status Upon Inclusion Scale (Points): Med. [Range]	Improvement as Defined in the Study n (%)
Myasthenia Gravis	21	56.71 ± 21.22	13/8	6.13 years	QMGS: 21 [15–31]	14 (66.6)
Guillain–Barré Syndrome	65	52.75 ± 14.13	26/39	14.7 days	Lovett scale: 2.5 [1–4]	34 (52.3)
Chronic Inflammatory Demyelinating Polyradiculo-neuropathy	30	60.37 ± 13.86	13/17	6.4 years	Lovett scale: 3 [2–4]	3 (10.0)
Neuromyelitis Optica spectrum disorders	12	49.75 ± 8.91	12/0	4.17 years	MRC score: 2 [2–5]	3 (25.0)
Autoimmune Encephalitis	13	61.85 ± 15.48	9/4	5.5 months	mRS: 3 [2–5]	4 (30.7)
Summary	141	56.28 ± 14.72	73/68	-	-	58 (41.13)

QMGS—Quantitative Myasthenia Gravis Score; mRS—modified Rankin Scale; MRC—Medical Research Council.

## Data Availability

Not applicable.
